# Straight versus S-shaped sternotomy: a histologic study in the sheep model

**DOI:** 10.1186/s13019-014-0173-x

**Published:** 2014-10-30

**Authors:** Bekir Inan, Fatih Kucukdurmaz, Sebnem Karakan, Melike E Teker, Caner Akcan, Gulay B Dilek, Kenan Daglioglu

**Affiliations:** Department of Cardiovascular Surgery, Faculty of Medicine, Bezmialem Vakıf University, Istanbul, Turkey; Department of Orthopedics and Traumatology, Bezmialem Vakıf University, Istanbul, Turkey; Department of Nephrology, Ankara Oncology Education and Research Hospital, Ankara, Turkey; Department of Cardiovascular Surgery, Malatya State Hospital, Malatya, Turkey; Department of CAE and Methodology Development, SDM Research and Engineering, Istanbul, Turkey; Department of Pathology, Ankara Oncology Education and Research Hospital, Ankara, Turkey; Department of Microbiology, Cukurova University, Adana, Turkey

**Keywords:** Sheep, Animal model, Coronary artery bypass, Cardiovascular surgery

## Abstract

**Introduction:**

Straight sternotomy is the most common access for open heart surgery. Techniques have been proposed for maximizing sternal stability in high-risk patients. This trend implies a growing need for newer surgical techniques. The aim of this experimental study in the sheep model is to evaluate median vs. S shaped sternotomy the feasibility of using a special device to accelerate the sternal instability and bone healing.

**Materials and methods:**

We enrolled 31 sheep, weighing 18–30 kg. For all animals a midline skin incision was made. In group I (n = 16 animals), straight median sternotomy and in group II (n = 15 animals), S-shaped incision was marked on the periosteum of the sternum by new created device for standard S-shaped sternotomy. Sternum biopsies were performed on second surgery month for all survived animals from the sternum and the surrounding soft tissue.

**Results:**

No early superficial wound complications were observed. Overall mortality in the initial approach group was 19.3% (6 sheep). In group I; 3 sheep had died on first surgery day, the reason may be hemorrhage and in group II; 3 sheep developed intractable VF during surgery procedure or immediately afterwards so died. There were statistically significant differences in the scores of bone healing between group 1 and group 2 (4.2 vs.7.3, ANOVA, p < 0.001).

**Discussion:**

Our work is based on the use of a standard S-shaped sternotomy procedure on sheep sternum. In our experience with the sternal healing in the sheep model, the process of new bone formation was accelerated with S- shaped cut than straight sternotomy procedure.

**Electronic supplementary material:**

The online version of this article (doi:10.1186/s13019-014-0173-x) contains supplementary material, which is available to authorized users.

## Background

Straightsternotomyis the most common access for open heart surgery [1],[2]. It usually heals well, but the incidence of sternal dehiscence has been reported between 0.5 and 8% as a consequence of mechanical breakdown and/or infection [3]-[6]. Sternal wound complications, including dehiscence and infection, remain challenging and occur in 1%–3% of patients undergoing cardiac surgery. This process leads to a variable mortality rate ranging from 14% to 47% [7]-[9]. Several techniques have been proposed for maximizing sternal stability in high-risk patients [10]-[15]. This trend implies a growing need for newer surgical techniques and devices to facilitate the operative procedures. In view of this trend, there is a great interest in the development of new methods and devices that may replace traditional sternotomy methods. Such devices will shorten the time needed to perform an anastomosis, and will significantly reduce wound and bone healing complications associated with the traditional anastomotic procedures. Thus, preclinical studies are essential for the development and validation of these new devices and procedures.

The use of the sheep as an animal model seemed to be ideal for simulating human cardiac surgical procedures due to the size of the chest cavity, which can accommodate devices and surgical instruments intended for human use.

The aim of this experimental study in the sheep model, is to evaluate median vs. S shaped sternotomy the feasibility of using a special device to accelerate the sternal instability and bone healing. This specific device (Sternum-S) was designed to create a Standard S-shaped sternotomy procedure.

## Methods

**Figure 1:**
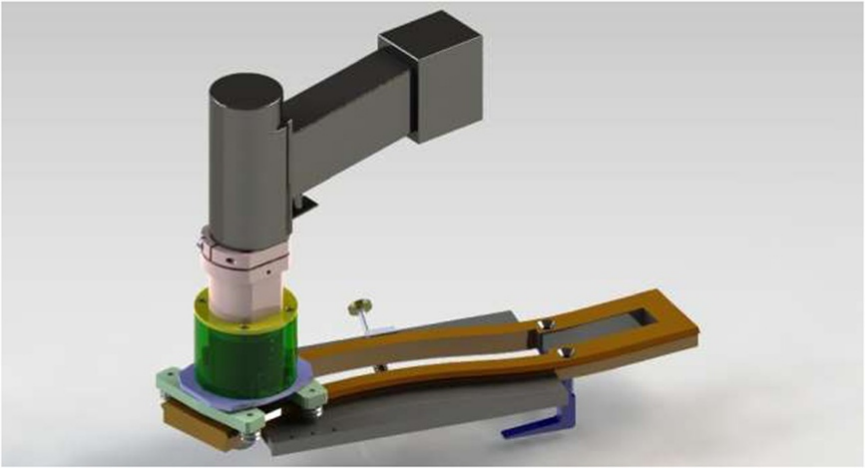
**Sternum S new created device for Standard S**-**shaped sternum incision.**

We enrolled 31 sheep (mean age: 2.5 ± 0.5 years), weighing 18–30 kg (mean, 23.5 ± 3.8 kg). Ethics approval was obtained through the animal care and ethics committee from Cukurova University. Following overnight fasting, animals were premedicated and were given a prophylactic dose of antibiotic (cefazolin 1 g i.v.). After induction with thiopental 10 mg/kg and intubation, an analgesic (buprenorphine 0.6 mg –0.01 mg/kg) was given i.m. before opening the chest, and an additional dose of 0.6 mg was given at the end of the procedure. After standard preparation, for all sheep a sternotomy was surgically created. The sheep was placed in a dorsal recumbence, and a median sternotomy from the tip of the xyphoid bone to the supra-sternal notch was performed. In group I (n = 16 animals), straight median sternotomy and in group II (n = 15 animals), a midline skin incision was made and S-shaped incision was marked on the periosteum of the sternum by new created device (Figure 1) for a Standard S-shaped. Care was taken to remain within the lateral confines of the manubrium and body of the sternum. Bleeding points of the periosteum and marrow were cauterized for hemostasis. No bone wax was used in either group only sutured with steel wire. Postoperative management of the sheep consisted of cefazolin injected intramuscularly at 1 g/day over five days. Analgesia was guaranteed using metamizol at 2 g intramuscularly every 12 h over five days. Sternum biopsies were performed on second surgery month for all survived animals from the sternum and the surrounding soft tissue and fixed in 4% (w/v) neutral buffered formalin pH 7.2–7.4 and sectioned to yield 3-μm-thick slices. The sections were stained with hematoxylin and eosin. The pathologist did the comparative evaluation as a blinded examiner. The over-bridging callus was graded histologically by a 10 point grading scale as described by Huddleston et al. [16]. Using a Olympus BX51 microscope, Olympus Optical Co., Ltd., Tokyo, Japan; grade 1: fibrous union; grade 2: predominantly fibrous tissue with some cartilage; grade 3: equal amounts of fibrous tissue and cartilage; grade 4: all cartilage; grade 5: predominantly cartilage with some woven bone; grade 6: equal amounts of cartilage and woven bone; grade 7: predominantly woven bone with some cartilage; grade 8: entirely woven bone; grade 9: woven bone and some mature bone; grade 10: lameller (mature) bone. All sheep were killed at second months after biopsy procedures.

Sternal instability was defined as a clicking sound, anteroposterior movement of the sternal edges elicited with mild pressure, and a discernible but small gap between the sternal edges. Sternal dehiscence was defined as complete separation of the two sternal halves with an open wound and/or frank mediastinitis. Superficial wound infection was defined as redness.

### Statistical methods

A statistical comparison was made between relevant parameters using Student’s t-test or x2-analysis as appropriate. A p-value of less than 0.05 was considered statistically significant.

## Results

No early superficial wound complications were observed. Overall mortality in the initial approach group (31sheep) amounted to19.3% (6 sheep, 3 sheep from group 1and 3 sheep from group 2). In group I; 3 sheep had died on first surgery day, the reason may be hemorrhage and in group II; 3 sheep developed intractable VF during surgery procedure or immediately afterwards so died. All surviving animals of the initial approach group, which were followed for more than two months, were taken biopsies from surgery area.In group 1, 7 developed superficial wound infection and 4 sheep had sternal instability which was detected at the 2 weeks follow-up; and 2 sheep had sternal dehiscence.

In group 2, among the survivors, 4 sheep developed superficial wound infection and 2 sheep developed sternal instability, sternal dehiscence developed in one sheep of the surviving sheep.

Woven and lameller bone formation and bone marrow organization was more obvious than group I. Cartilage was absent and a significantly decreased bone union rate had been consistently demonstrated in group I. Our results showed that group 2 (S-shaped sternotomy) had significant histologic differences in fracture healing as compared to group1 (straight sternotomy).

**Figure 2:**
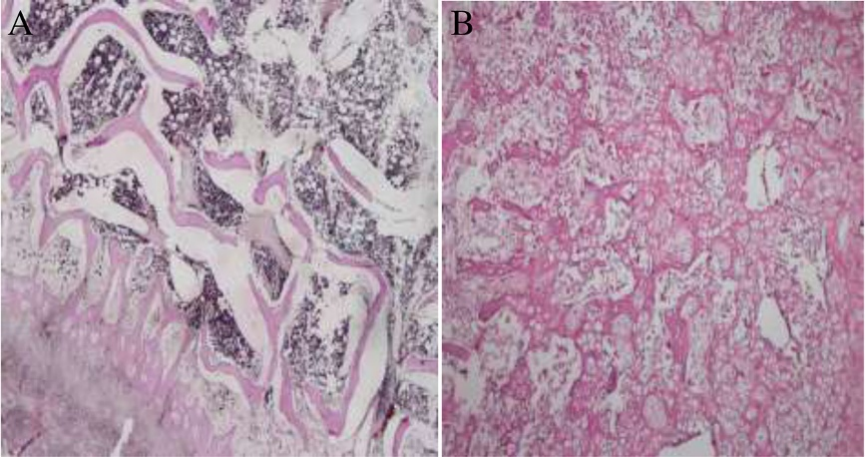
**There is a significant difference in the scores of bone healing between groups.** 2**A**: S-Shaped sternotomy; mature (lameller) bone formation (H & E x40), 2**B**: Straight shaped sternotomy; equal amounts of fibrous tissue and cartilage (H & E x100).

There were statistically significant differences in the scores of bone healing between group 1 and group 2 (4.2 vs.7.3, ANNOVA, *P*< 0.001). Group 1 and group 2 histological examples were depicted in Figure 2A and 2B.

## Discussion

A median sternotomy is a common approach to the heart during cardiac surgical procedures. The incidence of sternal bone healing complications have not changed since the 1980s, although major changes occurred in patients at risk, the population became older and sicker. Sternal instability or infection following median sternotomy is rare but important complications of median sternotomy. They are associated with prolonged hospitalization, increased hospital costs and high morbidity and even mortality [17]. Many references with median sternotomy appear every year in the cardiovascular literature and it is still a problem in the field of cardiac surgery. Most of these articles focus on the treatment of the complication, instead of the prevention on its appearance. Recent reports suggest that the process of healing may be improved by growth factors, which are small proteins, synthesized both by local cells at the injury site and by infiltrated blood inflammatory cells. These factors stimulate cell proliferation, migration, differentiation, and matrix synthesis and can affect chondrocyte metabolism, chondrogenesis, and improve bone healing.

The aim of this experimental study in the sheep model is to evaluate median vs. S shaped sternotomy the feasibility of using a special device to accelerate the sternal instability and bone healing.We tested a new designed device on sheep sternum by means of a similar procedure with human. In our experience with the sternal healing in the sheep model, the process of new bone formation was accelerated with S shaped cut than straight sternotomy procedure.

The technique of an S-shaped sternotomy was first described by Williams et al. who had used it for more than 2 years [18]. In our study, a reduction in the incidence of unstable sternum and sternal dehiscence was observed. Remarkably, bone healing parameters were statistically significant differences in the scores with S-shaped sternotomy.

Kucukdurmaz et al. found that interlocking sternotomy (a zigzag cut in three dimensions) is superior to those of the straight median sternotomy [19]. The zigzag cuts made the sternotomy line significantly more stable and provided more surface area for bony healing. We recommend an S-cut sternotomy as a safe technique with this special designed device that should be preferred in this subset in particular, as well as in general for all patients undergoing a sternotomy. The S-shaped of the sternal cut edges provides interlocking facets that fit into each other exactly and avoid vertical slippage between the two parts. Furthermore, the strong splint-like effect of the S-shaped sternotomy leads to firmer immobilization, promoting faster healing of the bone [20],[21]. On the histopathologic examination, group 1 showed fair amount of osteo-cartilaginous callus and union was nearly complete. Group 2 showed large amount of mature callus and union was mostly osseous. In group 1, cartilaginous and fibrous tissue was more prominent. Nevertheless, group 2 showed much higher stages of healing and lesser amount of fibrous tissue.

An understanding of this study of bone healing is critical to the consistent success of sternal fusion after S-shaped sternotomy. We documented that standard S-shaped sternotomy with this new device has great advances, it is only a faster osseous union that will ensure long-term sternal stability. Bone healing can greatly facilitate the potential for clinical success.

We wish to emphasize that one should exert caution and control while negotiating the curved part of the incision to remain well within the confines of the manubrium and body of the sternum. Use of force at these points may lead to excessive thinning or fractures in that region, which may be troublesome during closure.

## Conclusion

S-cut sternotomy made the sternotomy line more surface area and the sternal cut edges provides interlocking facets that fit into each other exactly. Bone healing formation was accelerated with S shaped cut than straight sternotomy procedure. Parameters were in the scores with S-shaped sternotomy. The S-shaped sternotomy was found to be a simple, safe, and reproducible technique which helps to reduce sternal instability and dehiscence.

## Authors’ original submitted files for images

Below are the links to the authors’ original submitted files for images. Authors’ original file for figure 1 Authors’ original file for figure 2

## Abbreviations

SDM: Name of company
TUBITAK: Scientific and Technological Research Council of Turkey
