# Supplementary figures and images for: Straight versus S-shaped sternotomy: a histologic study in the sheep model
Source: J Cardiothorac Surg. 2014 Oct 30;9:173. doi: 10.1186/s13019-014-0173-x (PMC4219003; doi:10.1186/s13019-014-0173-x)

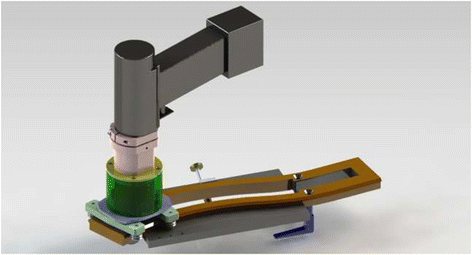

Supplement: Supplementary file 1 — Authors’ original file for figure 1 [file 13019_2014_173_MOESM1_ESM.gif]

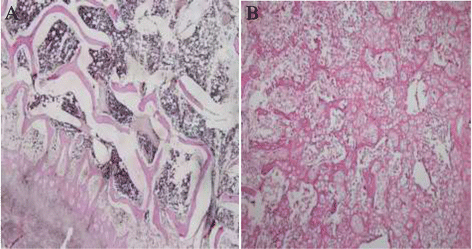

Supplement: Supplementary file 2 — Authors’ original file for figure 2 [file 13019_2014_173_MOESM2_ESM.gif]
